# Supplementary figures and images for: Cytoplasmic cyclin D1 modulates brain cortex development
Source: Cell Mol Life Sci. 2026 Apr 14;83(1):220. doi: 10.1007/s00018-026-06178-1 (PMC13187114; doi:10.1007/s00018-026-06178-1)

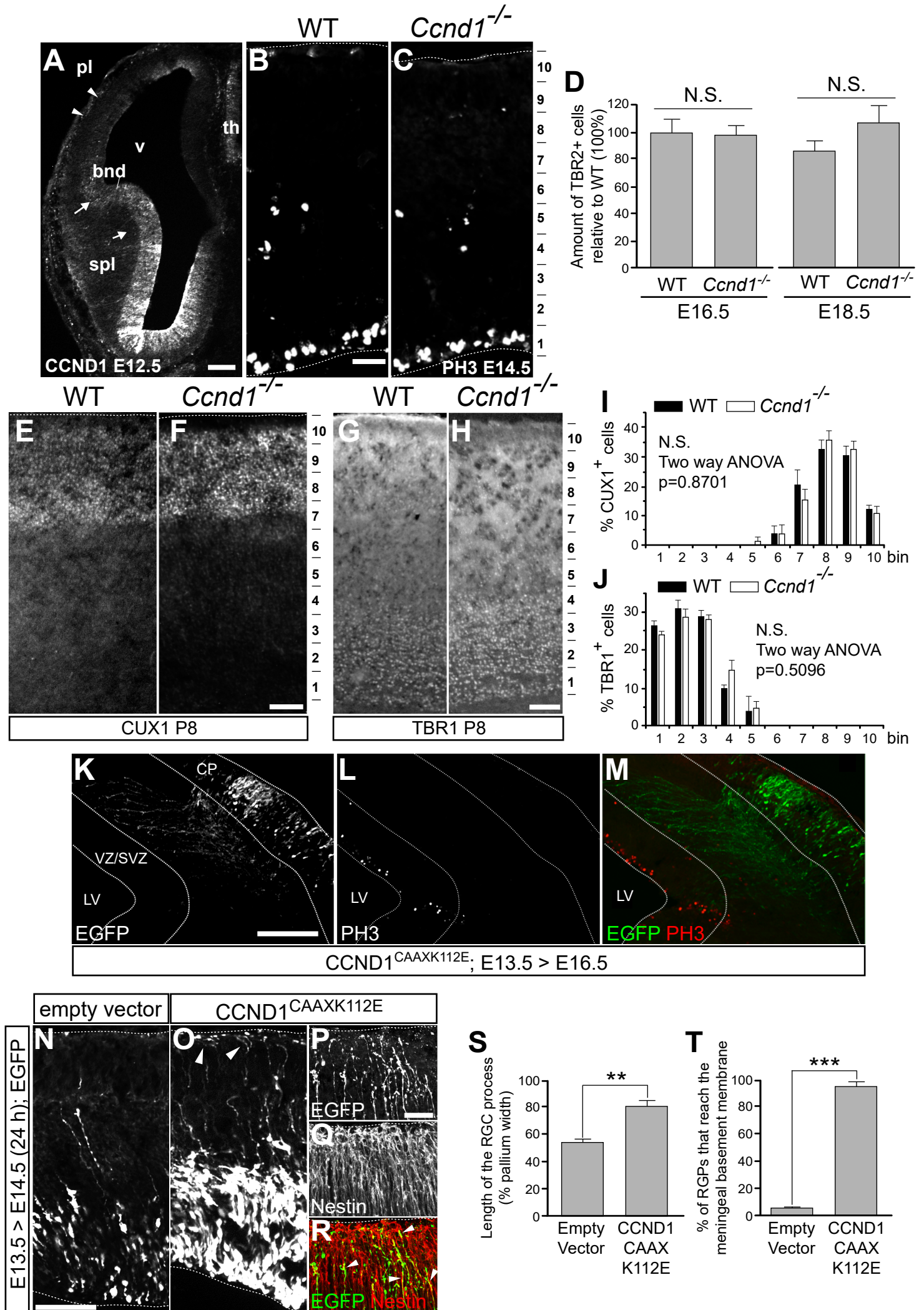

Supplementary Figure 1

Supplement: Supplementary file 1 — Supplementary file1 (PDF 8996 KB) [file 18_2026_6178_MOESM1_ESM.pdf]

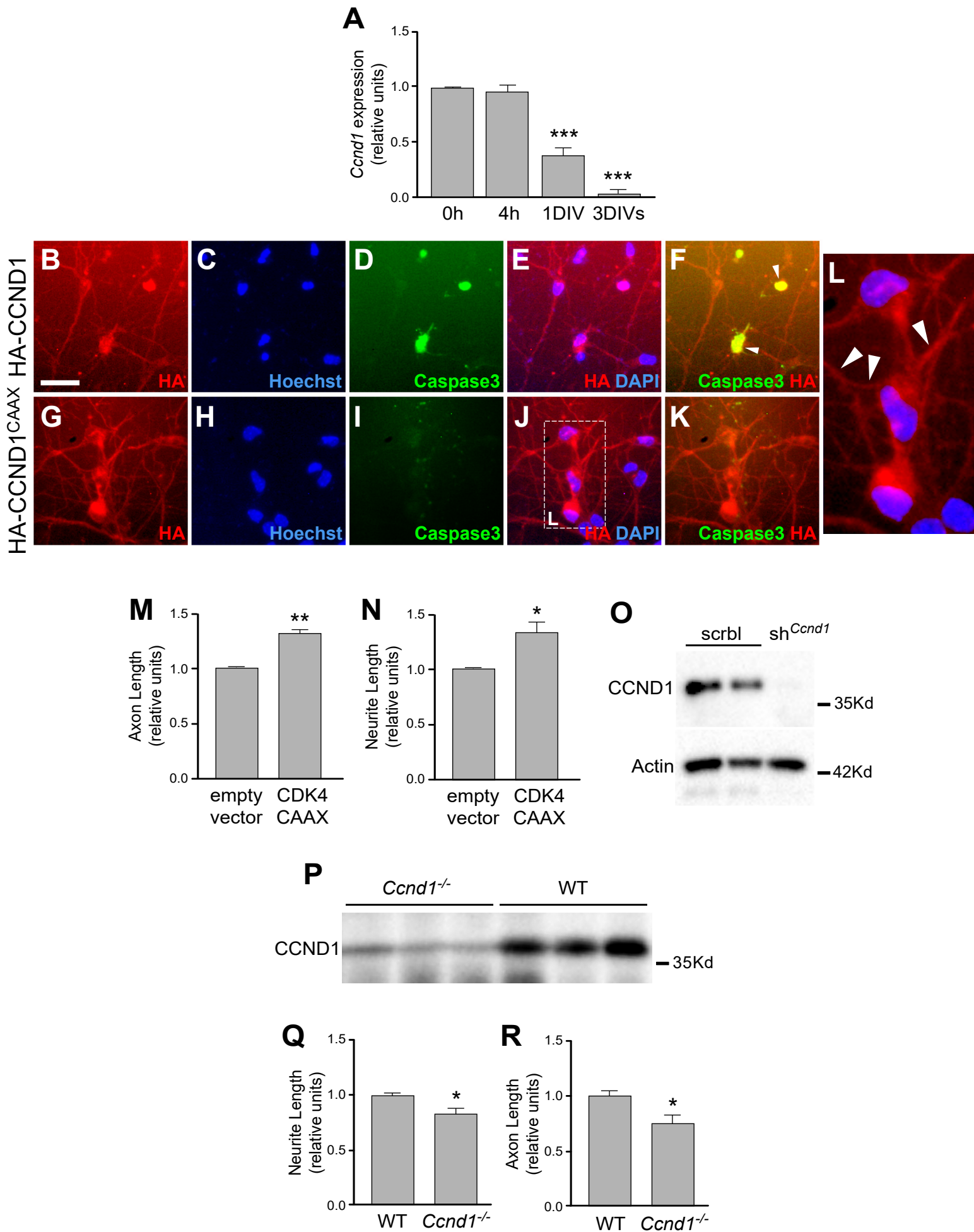

Supplementary Figure 2

Supplement: Supplementary file 2 — Supplementary file2 (PDF 4757 KB) [file 18_2026_6178_MOESM2_ESM.pdf]
